# Determination of oxidative stress markers and their importance in early diagnosis of uremia-related complications

**DOI:** 10.4103/0971-4065.50673

**Published:** 2009-01

**Authors:** V. Kolagal, S. A. Karanam, P. K. Dharmavarapu, R. D'Souza, S. Upadhya, V. Kumar, V. Kedage, M. S. Muttigi, J. K. Shetty, M. Prakash

**Affiliations:** Department of Biochemistry, Kasturba Medical College, Manipal, India

**Keywords:** Cardiovascular diseases, ceruloplasmin, copper, glutathione S-transferase, malonyldialdehyde, uremia

## Abstract

The existence of oxidative stress and the higher incidence of cardiovascular diseases in association with uremia is well proved. The uremic status of serum copper, ceruloplasmin (CP), protein thiols, malonyldialdehyde (MDA), and glutathione S-transferase (GST) levels was studied. The study was carried out on 51 chronic renal failure (CRF) patients who were not on hemodialysis therapy and on 42 healthy controls. Serum urea, creatinine, and MDA levels were found to be significantly increased (*P* < 0.001), and total protein, albumin, protein thiols, and copper levels were found to be significantly decreased in CRF patients compared to normal controls (*P* < 0.001). Ceruloplasmin levels were decreased significantly (*P* < 0.05), and there was no significant change in serum GST levels in CRF patients compared to normal controls. In conclusion, the significant increase in levels of MDA, and the decrease in levels of protein thiols, CP, and copper in uremia patients when compared to controls, reconfirms the presence of stress in this patient population. In view of the changes in other markers of oxidative stress, this absence of any significant change in the activity of GST in uremia patients compared to controls, warrants further study.

## Introduction

Enhanced oxidative stress has been well established in uremia.[1,2] Oxidative stress defines an imbalance between the formation of reactive oxygen species (ROS) and antioxidative defence mechanisms.[3] It has been proposed to play a role in cardiovascular (CVD) and infectious diseases, cancer, diabetes, anemia, and neurodegenerative pathology. The incidence of these diseases has been found to increase in uremia[4,5] with cardiovascular disease (CVD) being the major cause of death in patients affected by chronic renal failure (CRF).[6] The risk of CRF patients having a cardiovascular event has been reported to be 3–5 times higher than in the general population.[7,8] Nontraditional risk factors for CVD such as oxidative stress, are being given special emphasis not only to explain the high incidence of CVD, but also to identify new targets for therapeutic interventions.[6]

The production of ROS is a natural process; the phagocyte oxidant generation system which includes both polymorphonuclear neutrophils (PMNs) and monocyte-macrophage cells is based on the inducible production of ROS.[6,9] The phagocytes produce ROS to perform physiological processes, such as killing bacteria etc.[10] ROS and inflammation are deeply interrelated, as different oxidant free radicals are generated by phagocytic cells in response to inflammatory stimuli.[6] Transition metal ions such as iron or copper also contribute to excess ROS production through Fenton reaction.[3] ROS are further released together with proinflammatory cytokines, which in turn amplify oxidant generation.[11] The excess levels of ROS have been implicated in the damage to DNA, lipids, proteins, and the cell's carbohydrate content.[6] It may also affect the cells of host organisms, particularly at the sites of inflammation, contributing to proteinuria as observed in CRF patients.[1,12]

ROS are highly reactive compounds with a half-life of only seconds; therefore, their *in vivo* measurement to assess oxidative stress is generally not feasible. Instead, lipids, proteins, carbohydrate, and nucleic acid have lifetimes ranging from hours to weeks after being modified by ROS, which makes them ideal markers of oxidative stress.[13] The total thiol status in the body, especially thiol (-SH) groups present on protein are considered as major plasma antioxidants *in vivo* and most of the SH-groups are present over albumin and are major reducing groups present in our body fluids.[14,15] Ceruloplasmin (CP) is an alpha-2, acute phase-responsive, multicopper oxidase glycoprotein that contains >95% of the copper present in plasma. Antioxidant activity of CP can be ascribed mainly to its ferroxidase activity, which inhibits ferrous ion-stimulated lipid peroxidation and the formation of hydroxyl radicals in the Fenton reaction, and is also a scavenger of ROS.[16,17]

Copper is a transition metal ion and the great bulk of copper in the body is bound to storage and transport proteins in forms that are unable to act as pro-oxidants. Surprisingly, adequate copper is required to maintain antioxidant defenses within the body, and it has been demonstrated that copper deficiency in animals can have pro-oxidant effects.[18] During lipid peroxidation, unstable hydroperoxides resulting from peroxyl radical-dependent chain reactions involving unsaturated fatty acyl moieties later break down to smaller and more stable products like malonyldialdehyde (MDA) or thiobarbituric acid-reactive substances (TBARS), which are considered to be oxidative stress markers.[6] Glutathione S-transferase (GST) comprises a multigene family of proteins involved in the metabolism of many disease-causing electrophilic substrates and it protects the cells against oxidative stress.[19] It also plays a role in the detoxification of organic hydroperoxides.[20] Different GST subclasses are localized to specific parts of body.[21] α-GST is localized to specific parts of the renal tubule (proximal) and is readily released into the urine during injury, therefore, it is considered to be an excellent biomarker for proteinuria.[22]

In the present study, we aim to assess oxidative stress by determining relevant antioxidant levels and oxidative stress markers in CRF patients compared to normal controls.

## Materials and Methods

### Subjects and samples

The study was carried out on 51 CRF patients who were not on hemodialysis therapy and on 42 healthy controls. The causes of CRF were: chronic glomerulonephritis (30 cases), diabetic nephropathy (16 cases), and unknown (five cases), and all of them were on conservative management. None of the patient groups received any form of antioxidant medication; all of them were on renal diet (50g protein and 5g salt/day). The healthy controls were not on any kind of prescribed medication or dietary restrictions. Informed consent was taken from all subjects involved in the study and the study was approved by the Institutional Review Board. Blood samples (5mL) were drawn into plain vacutainers from the antecubital veins of healthy controls and CRF patients. The blood was allowed to clot for 30min and centrifuged at 2000 *g* for 15min for clear separation of serum.

### Biochemical determinations

Special chemicals such as 5', 5'-dithio-*bis* (2-nitrobenzoic acid) (DTNB), 1-chloro-2, 4-dinitrobenzene (CDNB), reduced glutathione (GSH), bathocuprine disulphonate disodium salt (BCDS), and *p*-phenyl diammonium dichloride (PPD) were obtained from Sigma Chemicals, St Louis, MO, USA. All other reagents were of chemical grade.

#### Thiol assay:

The reaction mixture containing 900 μL 2 mM Na_2_EDTA in 0.2 M Na_2_HPO_4_, 20 μL 10 mM DTNB in 0.2 M Na_2_HPO_4_, and 100 μL serum was incubated at room temperature for five min; the absorbance was read at 412 nm. Appropriate sample and reagent blanks were prepared simultaneously and the respective absorbance was noted. Corrected absorbance values were used to calculate serum protein thiol content using a molar extinction coefficient of 1600/M/cm and values were expressed as μM. The calibration curve was produced using GSH dissolved in phosphate-buffered saline (PBS).[23]

#### GST assay:

One mL of reaction mixture containing 850 μL of 0.1M phosphate buffer, pH 6.5; 50 μL 20 mM CDNB (1-chloro 2,4-dinitrobenzene), and 50 μL 20 mM GSH was preincubated at 37° C for 10 min. Reaction was started by adding 50 μL serum and GST activity was assayed kinetically. Reaction was followed at one minute intervals for five minutes by measuring the absorbance at 340 nm. GST was determined by using a molar extinction coefficient of 9.6/mM/cm and GST activity was expressed as IU/L.[24,25]

#### MDA assay:

The reaction mixture containing 1 mL 0.67% thiobarbituric acid (TBA), 500 μL 20% tricarboxylic acid (TCA), and 100 μL serum was incubated at 100° C for 20 min and centrifuged at 12,000 rpm for 5 min. The absorbance of the supernatant was read at 532 nm and MDA concentration was determined by using a molar extinction coefficient of 1.56 × 10^5^/M/cm and the values were expressed as μM.[26]

#### Ceruloplasmin assay:

The reaction mixture containing 3 mL PPD and 60 μL plasma was incubated at 37°C for 15 minutes. After incubation, 600 μL of 3% sodium azide was added and the absorbance was read at 546 nm. An appropriate sample blank was prepared simultaneously and its absorbance was noted. Plasma ceruloplasmin levels were calculated as (absorbance of test – absorbance of blank) × 237, and values were expressed as mg/dL.[27]

#### Copper assay:

Reaction mixture was composed of 200 μL 6M guanidine hydrochloride containing 0.1M ascorbic acid in 0.1M acetate buffer, pH 4.8, and 100 μL plasma. After incubation for 10 minutes at room temperature, 200 μL 0.89 mM BCDS in 2M Tris was added and mixed by vortexing. The absorbance was read at 480 nm, as was that of appropriate sample and reagent blanks that were prepared simultaneously. Corrected absorbance values were used to calculate plasma copper levels using a molar extinction coefficient of 2.388 × 10^3^/M/cm. Plasma copper values were expressed as μg/dL.[28]

#### Parameters determined using automated assays:

In CRF patients and healthy controls, serum total protein levels were determined using Biuret method, albumin level was measured by the bromocresol green dye-binding method, urea by the urease-glutamate dehydrogenase method, and creatinine by Jaffe's method using a Clinical Chemistry Autoanalyzer (Hitachi 912).

### Statistical analysis

The results were expressed as mean ± standard deviation (SD). *P* < 0.05 was considered to be statistically significant. Statistical analysis was performed using the Statistical Package for Social Sciences (SPSS-13, Chicago, USA). Independent sample t-test was used to compare mean values and Pearson's correlation was applied to correlate the parameters.

## Results

**Figure 1:**
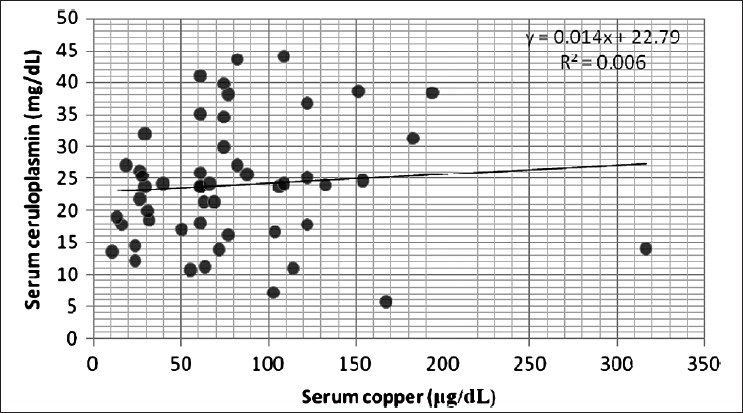
Correlation between serum copper and ceruloplasmin

**Figure 2:**
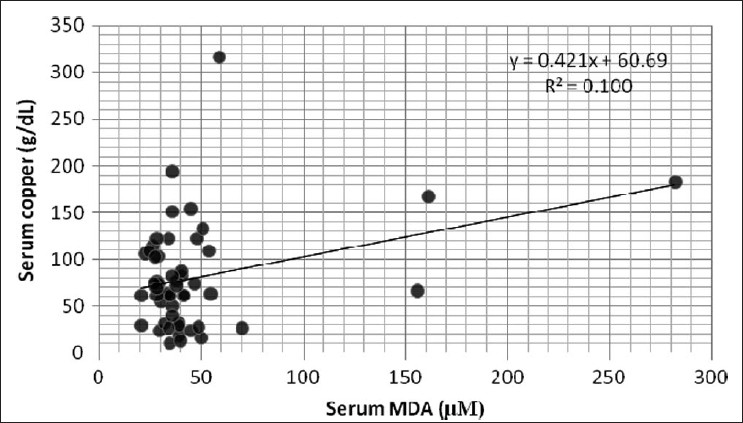
Correlation between serum copper and MDA

As shown in Tables 1 and 2, serum urea, creatinine, and MDA levels were found to be significantly increased (*P* < 0.001), whereas total protein, albumin, protein thiols, and copper levels were found to be significantly decreased in CRF patients compared to normal controls (*P* < 0.001). Ceruloplasmin levels had decreased significantly (*P* < 0.05) and there was no significant change in serum GST levels in CRF patients compared to normal controls. On applying Pearson's correlation, serum copper correlated negatively with urea (r=−0.491, *P* < 0.01), and creatinine (r=−0.496, *P* < 0.01); but positively with total protein (r=0.573, *P* < 0.01), albumin (r=0.601, *P* < 0.01), protein thiols (r=0.595, *P* < 0.01), ceruloplasmin (r=0.381, *P* < 0.01) [Figure 1], and MDA (r=0.342, *P* < 0.01) [Figure 2].

**Table 1:** Demographic and clinical characteristics of chronic renal failure patients and normal controls (mean ± SD)

Characteristics	Normal controls (*n* = 42)	Chronic renal failure (*n* = 51)
Age (years)	50 ± 14	49 ± 15
Sex (M/F)	37/5	43/8
Serum urea (mM)	11.09 ± 2.09	31.14 ± 17.59*
Serum creatinine (μM)	85.81 ± 17.03	385.14 ± 226.98*

* *P* < 0.001 compared to normal controls

**Table 2:** Serum oxidative markers and antioxidants in normal controls and patients with chronic renal failure (mean ± SD)

	Normal controls (*n* = 42)	Chronic renal failure (*n* = 51)
Total protein (g/dL)	7.5 ± 0.48	5.9 ± 0.83*
Albumin (g/dL)	4.77 ± 0.51	3.14 ± 0.81*
Protein thiols (μM)	366.61 ± 57.08	223.11 ± 64.97*
Copper (μg/dL)	102.61 ± 21.37	53.94 ± 23.93*
Ceruloplasmin (mg/dL)	28.04 ± 5.56	23.91 ± 9.57**
GST (IU/L)	1.17 ± 0.53	1.19 ± 0.48
MDA (μM)	3.6 ± 1.98	46.63 ± 42.34*

* *P* < 0.001 compared to normal controls

** *P* < 0.05 compared to normal controls

## Discussion

Proteinuria is a well established finding in CRF patients.[29] Total protein and albumin levels in serum were decreased in these patients when compared to normal controls. Previous studies have shown significantly decreased protein thiols in the serum of uremia cases which correlated positively with serum albumin.[30] In agreement with previous studies, our study also shows decreased protein thiols in CRF patients. Oxidants could induce oxidative damage to plasma proteins, giving rise to advanced oxidation protein products (AOPP), levels of which have been reported to be significantly high in CRF patients.[2] AOPPs are potential uremic toxins and mediators of inflammation.[31] Furthermore, serum levels of AOPPs increase with progressive renal failure and are intimately associated with CVD.[32] A previous study has shown low levels of protein thiols, which correlates negatively with levels of AOPPs, a condition termed as “thiol stress” that contributes to the pathogenesis of CVD in this patient population.[14] In addition, “thiol stress” may be quantitatively important due to its reversible and treatable nature in CRF patients.[14]

CRF is an inflammatory condition and we hypothesized that the levels of CP, an acute phase protein, should increase in these patients. In contrast to our hypothesis, we observed that levels of CP decreased significantly in CRF patients as compared to the controls. Possible factors contributing to this observation were proteinuria and damage to this excreted protein due to the prevalent oxidative stress. Advanced glycation end products (AGEs) are formed during nonenzymatic, glyco-oxidation reactions involving plasma proteins. These reactions are enhanced in uremia where there is increased concentration of many α-oxaldehydes such as glyoxal, methylglyoxal etc. Like AOPPs, AGEs are also a class of uremic toxins.[33–35] ROS may disrupt copper binding to CP, thereby impairing its normal protective function while liberating the copper.[36] Thus, formation of uremic toxins and damage due to ROS may decrease CP levels in CRF patients. This observation supports our study which suggests that levels of plasma copper correlate positively with CP as shown in Figure 1. The free copper may bind loosely to albumin and other available low molecular weight substances that have been filtered by the diseased kidney and excreted into urine.

Before being excreted in urine, free copper may participate in metal ion-catalyzed Fenton and/or Haber-Wiess reactions to generate ROS. Some of the events associated with copper toxicity in uremia may be due to the generation of ROS by metal ions such as copper. Copper ions display high affinity for thiols and amino groups occurring in proteins. This indiscriminate binding may damage the structure of proteins, thus modifying their biological functions.[37] Copper-dependent oxidation of LDL was effectively inhibited by thiol groups.[38] Our study demonstrated that protein thiols are sacrificed to quench ROS which are produced excessively in CRF patients, thus the levels of these protein thiols decrease in these patients. Increased availability of free copper may oxidize available thiols, which in turn, decreases the protective activity of thiols for the suppression of metal ion-dependent LDL oxidation.

Other authors have suggested that serum copper, determined by TBARS levels, is the major determinant of serum lipid peroxidation status.[39] Our study is consistent with these other studies in that there is a positive correlation between serum copper and MDA as shown in Figure 2. Thus, we propose that the decrease in the level of CP-bound copper and the consequent enhanced availability of loosely bound and/or free copper ions may contribute to increased formation of MDA. Thus, our study highlights the possible role of copper-mediated ROS generation which contributes to aggravation of CRF-related complications and hence, the importance of its determination in these patients.

As GST is involved in the detoxification of electrophiles and hydroperoxides, it is generally expected that its plasma levels should have increased in CRF patients due to enhanced oxidative stress. In contrast, we found no significant difference in the serum levels of GST in CRF patients when compared to healthy controls. A previous study described inhibition of RBC–GST activity with uremic plasma.[40] Hence, there is a possibility that the presence of substances in uremic plasma (possibly uremic toxins) may contribute to decreased GST activity. Thus, we postulate that in early stages of uremia, the serum levels of GST may have increased due to its detoxification property, but with the progress of the disease and the associated accumulation of uremic toxins, GST levels may gradually decrease so that at the time of determination, GST levels were similar to those in healthy controls. However, this observation and the associated hypothesis warrant further study.

In conclusion, our study reconfirms the observations of earlier studies, but with a different patient population in a different geographical location. We have observed increased uremic stress in CRF patients, which may be induced by metal ion. In agreement with previous studies, we found a deficiency of antioxidant levels in CRF patients, a finding that emphasizes the importance of antioxidant supplementation in these patients. In contrast to previous studies, we did not observe any change in activity of GST in CRF patients, a finding that warrants further study in different patient populations and with other experimental designs.
